# Rapid and Cost-Effective Diagnostic Blot Assays Based on the Use of Plant-Produced Recombinant Antigens: Lessons Learned from the SARS-CoV-2 RBD Antigen

**DOI:** 10.3390/ijms26104500

**Published:** 2025-05-08

**Authors:** Chiara Miele, Dania Ramadan, Leonardo Lupacchini, Carla Marusic, Valeria D’Argenio, Maria Giovanna Valente, Antonella Spila, Gianluca Gessoni, Veronica Alfano, Patrizia Ferroni, Marcello Donini, Fiorella Guadagni

**Affiliations:** 1 Department for the Promotion of Human Sciences and Quality of Life, San Raffaele Roma Open University, 00166 Rome, Italy; chiara.miele@sanraffaele.it (C.M.); valeria.dargenio@uniroma5.it (V.D.); fiorella.guadagni@uniroma5.it (F.G.); 2 InterInstitutional Multidisciplinary Biobank (BioBIM), IRCCS San Raffaele, 00166 Roma, Italy; dania.ramadan@sanraffaele.it (D.R.); maria.valente@sanraffaele.it (M.G.V.); antonella.spila@sanraffaele.it (A.S.); veronica.alfano@sanraffaele.it (V.A.); 3 Laboratory of Molecular and Cellular Neuroscience, IRCCS San Raffaele, 00166 Rome, Italy; leonardo.lupacchini@sanraffaele.it; 4 Division of Biotechnology, ENEA Casaccia Research Center, 00123 Roma, Italy; carla.marusic@enea.it (C.M.); marcello.donini@enea.it (M.D.); 5 CEINGE-Biotecnologie Avanzate Franco Salvatore, 80145 Napoli, Italy; 6 Transfusion Medicine Department of Venezia, Ospedale dell’Angelo-Mestre, 30173 Venezia, Italy; gianluca.gessoni@aulss3.veneto.it; 7 San Raffaele Cassino, 03043 Cassino, Italy

**Keywords:** diagnostic tests, plant-based molecular farming, recombinant antigens, SARS-CoV-2

## Abstract

The ongoing demand for reliable, cost-effective, and scalable diagnostic solutions during the COVID-19 pandemic emphasized the need for innovative production platforms. In this study, we present a plant-based molecular farming (PMF) strategy for the production of the receptor-binding domain (RBD) of the SARS-CoV-2 spike protein fused with an Fc region (RBDw-Fc). The RBDw-Fc antigen was transiently expressed in the *Nicotiana benthamiana* plant, achieving high yields and purity. Its functionality was assessed through antigen–antibody binding assays. The purified antigen was subsequently employed in the development of a rapid diagnostic blot assay capable of screening plasma EDTA samples from pre- and post-vaccinated as well as pre- and post-infected individuals, demonstrating high sensitivity and specificity. Our results show that the RBDw-Fc-based assay is effective for SARS-CoV-2 detection and offers considerable advantages in terms of production speed, scalability, and cost efficiency compared to traditional systems, such as cell-culture-based production. The assay delivers accurate results in just a few minutes, making it particularly suitable for clinical and resource-limited settings. This study highlights the versatility of PMF as a platform for producing high-quality reagents, with promising applications beyond SARS-CoV-2 diagnostics. The RBDw-Fc antigen-based method provides a model for the rapid, economical, and flexible development of screening tools for emerging infectious diseases and future pandemics.

## 1. Introduction

Plants can be successfully used as “biofactories” for producing complex proteins with therapeutic functions, such as growth regulators, antibodies, vaccines, hormones, cytokines, and enzymes, or for diagnostic purposes. This is primarily due to their ability to perform post-translational modifications that are often essential for maintaining protein functionality [1]. This area of plant biotechnology, known as “Plant Molecular Farming” (PMF), represents an innovative and advantageous biopharmaceutical production strategy. It offers significant benefits, including reduced production costs, increased speed, and enhanced biosafety [2,3,4,5].

During the COVID-19 pandemic, the global demand for diagnostic kits rapidly increased, leading to shortages of reagents such as recombinant antigens and antibodies. This highlighted the need for alternative platforms capable of producing biopharmaceuticals at scale [6]. As a result, both the scientific community and the biopharmaceutical industry have shown increased interest in exploring the clinical feasibility and scalability of PMF.

PMF has already demonstrated its effectiveness in producing antigens for the SARS-CoV-1 virus [7], and more recently, several studies have reported the successful use of plants to express recombinant antigens for SARS-CoV-2, not only for vaccine development but also for diagnostic applications [8,9,10,11].

Currently available diagnostic tests for SARS-CoV-2 vary in format, with many relying on recombinant antigens or monoclonal antibodies. Antigen-based tests, commonly referred to as antibody tests, detect the presence and levels of IgM and IgG in blood, serum, or plasma, thereby determining whether an individual has been exposed to the virus. These tests employ recombinant antigens that mimic the virus and bind to any specific antibodies in the sample [12]. While effective, the production of these reagents is often time-consuming and costly. Therefore, alternative strategies for producing high-quality and low-cost reagents are critical to ensure rapid responses to future health emergencies.

In this study, we describe the use of plant-produced recombinant antigens for diagnostic purposes. Specifically, we tested the receptor-binding domain (RBD)w-Fc antigen of the SARS-CoV-2 spike protein [13] and employed it to develop an antigen-based assay capable of screening serum samples from vaccinated or infected individuals. Our results demonstrate that this PMF-based approach is not only effective for rapid SARS-CoV-2 detection but also provides a scalable and cost-efficient method that can be applied to other diseases, thereby improving the production of high-quality diagnostic reagents on a large scale.

## 2. Results

### 2.1. Plant Production of the RBDw-Fc Recombinant Antigen

**Figure 1:**
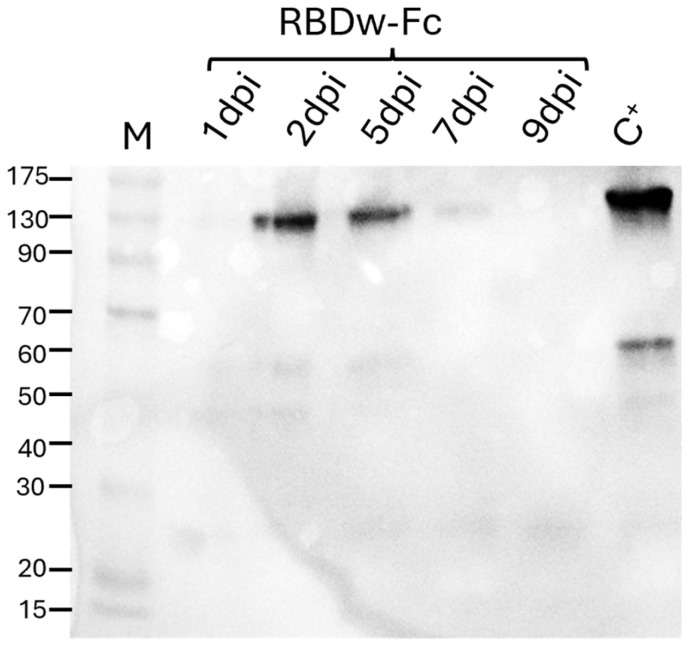
Western blot analysis of plant-expressed RDBw-Fc. 12% SDS-PAGE analysis under non-reducing conditions followed by Western blotting using anti-SARS-CoV-2 plant-produced mAb675 as the primary antibody. RBDw-Fc 1, 2, 5, 7, and 9 dpi: days after agroinfiltration (20 μL of extract). C+: commercial RBD protein (125 ng). M: molecular weight marker.

Six weeks after sowing, *N. benthamiana* plants were used for agroinfiltration using a vacuum pump. These were entirely immersed in the bacterial suspension containing, in an equimolar concentration (1:1 ratio), the cultures transformed with the constructs coding for RBDw-Fc and the gene silencing inhibitor P19. Leaf samples were collected at 1, 2, 5, 7, and 9 days post-infiltration (dpi) to identify the day corresponding to the peak of expression of the recombinant antigen (Figure 1).

Western blot analysis performed under non-reducing conditions, using a human neutralizing SARS-CoV-2 monoclonal antibody (mAb 675) against RBD, detected the presence of a band at about 110 kDa, which corresponds to the RBDw-Fc protein in dimeric form, also visible in the commercial RBD protein used as a positive control (C+), and other faint bands at lower molecular weight, probably due to the unassembled monomeric antigen (60 kDa) and to degradation fragments (Figure 1). The RBDw-Fc showed the maximum level of accumulation 2 days after agroinfiltration.

### 2.2. RBDw-Fc Purification and Characterization

**Figure 2:**
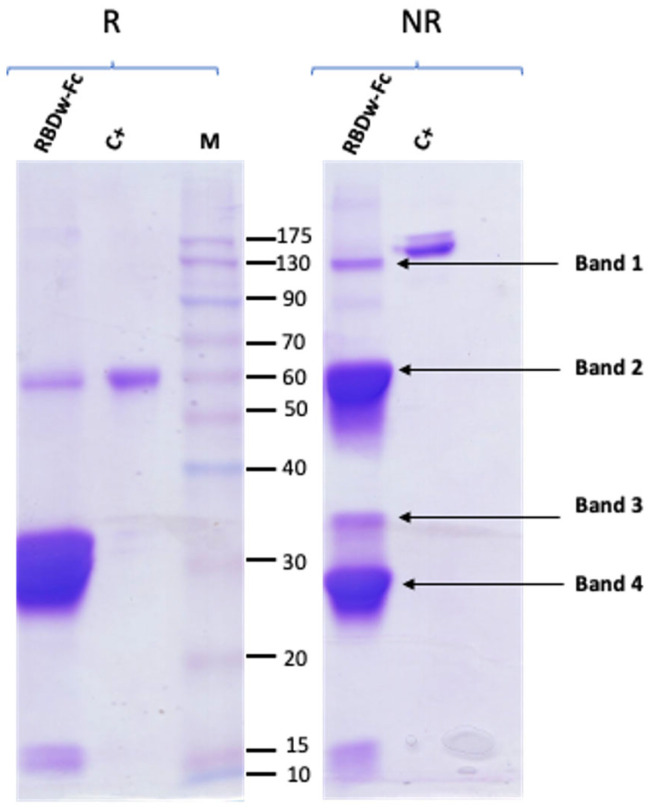
Analysis of purified RBDw-Fc. 12% SDS-PAGE under reducing (R) and non-reducing (NR) conditions, followed by Coomassie staining. In the left (R) and right (NR) panels, 10 µg of RBDw-Fc and 2 µg of the commercial RBDw-Fc (C+) were loaded. In the right panel (NR): band 1: intact dimeric RBDw-Fc. Bands 2, 3, and 4: protein degradation fragments.

Purification of the RBDw-Fc antigen was performed by affinity chromatography. Starting from a total extract obtained by trituration and mechanical homogenization of the leaves (40 g), the RBDw-Fc antigen was purified by HiTrap protein-A affinity column chromatography, as described in Section 4. The protein was then analyzed by 12% SDS-PAGE analysis and Coomassie staining under reducing (R) and non-reducing (NR) conditions (Figure 2).

Two bands were identified in the reducing gel, one relating to the intact antigen in monomeric form (55 kDa) and a very intense band with a molecular weight of 30 kDa. In the non-reducing gel, three main bands were highlighted: a first band of expected molecular weight around 110 kDa corresponding to the intact antigen in dimeric form, and two other more intense bands at lower molecular weights, indicating the degradation of purified RBDw-Fc (Figure 2). The purification yield calculated on the basis of three separate experiments was found to be 45.8 ± 15.8 mg per kg of agroinfiltrated leaves. This result is in line with previous experiments [13] in which the obtained yields were 35.8 ± 5.8 mg per kg of leaves.

Thus, the strategy used herein seems to be effective for fast RBDw-Fc antigen production with good efficiency based on the obtained yield.

### 2.3. The RBDw-Fc Antigen-Based Test Development and Validation

**Figure 3:**
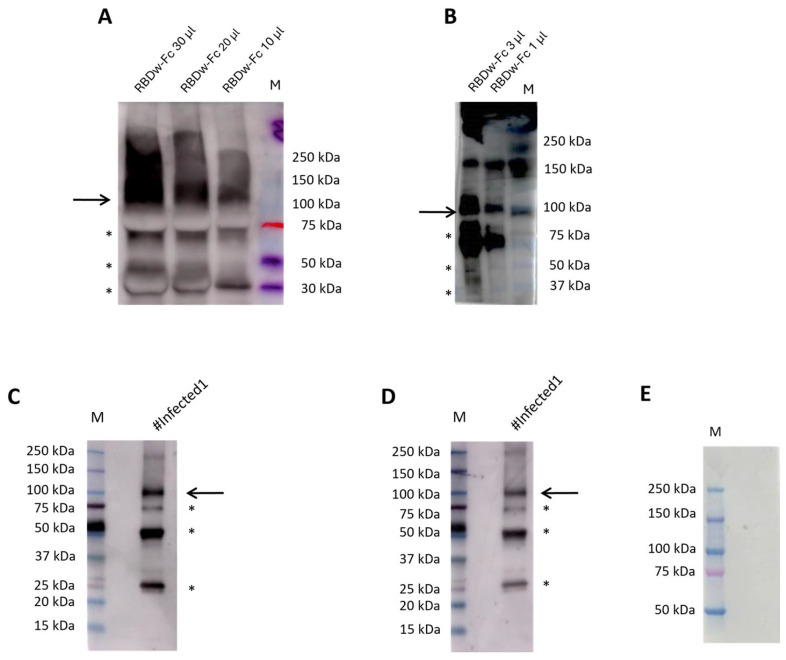
Western blot analysis using decreasing concentrations of RBDw-Fc antigen. (**A**) RBDw-Fc antigen at 30 µg/µL, 20 µg/µL, 10 µg/µL, hybridized with a known post-infection plasma EDTA sample (#Infected1) diluted 1:500 in 5% milk. Loading Marker (M) BenchMark™ Pre-stained Protein Ladder. (**B**) RBDw-Fc antigen at 3 µg/µL and 1 µg/µL, hybridized with a known post-infection plasma EDTA sample (#Infected1) diluted 1:500 in 5% milk. Loading Marker (M) Kaleidoscope Bio-Rad. (**C**) RBDw-Fc antigen at 1 µg/µL, hybridized with a known post-infection plasma EDTA sample (#Infected1) diluted 1:1000 in 5% milk. Loading Marker (M) Kaleidoscope Bio-Rad. (**D**) RBDw-Fc antigen at 1 µg/µL, hybridized with a known post-infection plasma EDTA sample (#Infected1) diluted 1:2000 in 5% milk. Loading Marker (M) Kaleidoscope Bio-Rad. (**E**) RBDw-Fc antigen at 1 µg/µL, hybridized with goat anti-human IgG secondary antibody dilution 1:10,000 in 5% milk. Loading Marker (M) Kaleidoscope Bio-Rad. * Indicates a degradation product; arrows indicate the dimeric form of the antigen.

For the development of the method, we analyzed three plasma EDTA, three plasma citrate, three lithium heparin plasma, and three serum-matched samples obtained from three patients with a confirmed diagnosis of COVID-19, each used as an antibody against a batch of plant extracts containing the RBDw-Fc product in *Nicotiana benthamiana*. In particular, we improved the conditions for this type of sample as described below. To determine the optimal conditions of RBDw-Fc antigen functionality, we performed a Western blot analysis by loading decreasing concentrations of the antigen (30 µg/µL, 20 µg/µL, 10 µg/µL, 3 µg/µL, and 1 µg/µL), under non-reducing (NR) conditions and hybridizing them with all 12 SARS-CoV-2 positive samples diluted 1:500 in 5% milk overnight at 4 °C (Supplementary Figure S1A,B). The results showed that the best antigen concentration was 1 µg/µL (Figure 3A,B).

Moreover, the best result was obtained using EDTA samples with an antigen concentration of 1 µg/µL. Since the results with serum and EDTA were comparable, and we had many more EDTA samples than serum samples, we chose to use this sample type. This approach allows for the use of a small sample volume; additionally, plasma EDTA is versatile enough to be utilized for different purposes, such as hematology testing, blood group typing, genetic, and molecular testing, which makes a single blood draw suitable for multiple tests. Furthermore, we verified two other batches of the same antigen, obtaining similar results (Supplementary Figure S2). These findings demonstrate that antigens produced in plants are characterized by high quality and efficiency.

Next, to further improve antigen–antibody binding efficiency, we tested three different dilutions of plasma EDTA in 5% milk (1:500, 1:1000, and 1:2000) overnight: interestingly, we found that the 1:2000 dilution yielded the best results (Figure 3B–D).

A band corresponding to a molecular weight of 110 kDa (indicated with an arrow in Figure 3) was clearly visible in the non-reducing gel (Figure 3A–D), representing the dimeric form of the antigen. Additional bands were observed at molecular weights of 70 kDa, 50 kDa, and 25 kDa (indicated with asterisks in Figure 3), representing degradation products, as previously reported [13]. To exclude the possibility of non-specific binding between the antigen and the human IgG recognized by the secondary antibody, we incubated the antigen only with the secondary antibody for 1 h. As expected, no bands were detected (Figure 3E). These results demonstrate the high specificity and binding affinity between our RBDw-Fc antigen and the IgG in plasma EDTA samples, supporting the efficacy of the proposed test and its potential use in clinical settings.

### 2.4. RBDw-Fc Antigen-Based Test for SARS-CoV-2 Infection Rapid Screening

After proving the ability to obtain efficient production of the RBDw-Fc antigen in a PMF context and the subsequent optimization and validation of the analytic procedures using different human samples, we aimed to verify the possibility of using this proposed strategy for the rapid screening of SARS-CoV-2 infection.

**Figure 4:**
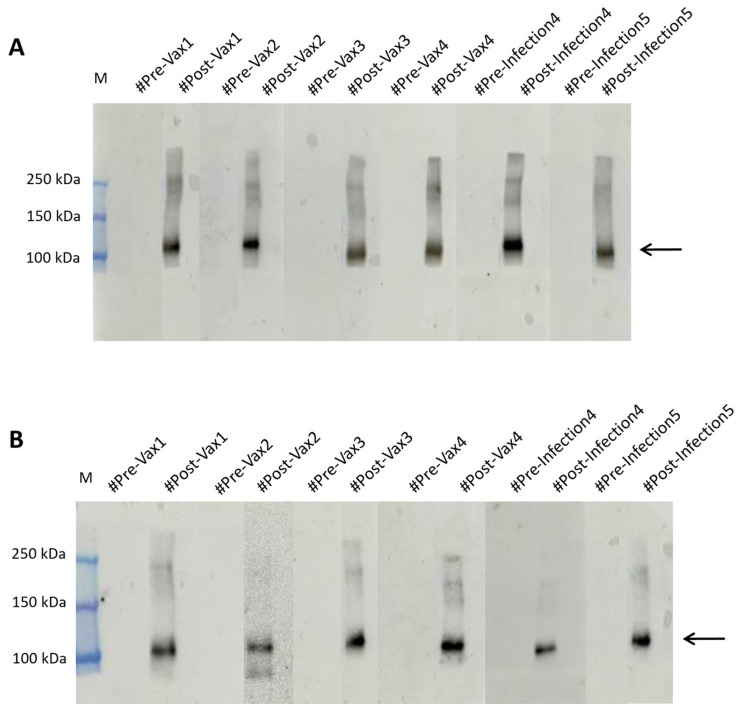
Western blot analysis of EDTA plasma samples for the spike SARS-CoV-2 protein. This figure represents the blind analysis of plasma EDTA samples. Negative samples show no band, while positive samples show the 110 kDa band (indicated by an arrow). RBDw-Fc antigen in the amount of 1 µg was loaded into each lane. (**A**) The membranes were incubated with plasma EDTA as a primary antibody overnight, and detection was performed with goat anti-human IgG F(ab’)2 antibody conjugated with HRP, incubated for 1 h. (**B**) The individual membranes were incubated for 10 min with plasma EDTA as primary antibody and then for 10 min with goat anti-human IgG F(ab’)2 antibody conjugated with HRP.

To this aim, we collected 98 anonymous samples, including either pre- and post-vaccination or pre- and post-infection samples, from the Interinstitutional Multidisciplinary BioBank (BioBIM) of the IRCCS San Raffaele in Rome, Italy (Supplementary Table S1). All samples were blind tested on membranes where the produced antigen had been run and were then hybridized overnight at 4 °C to detect the presence or absence of bands, as shown in Figure 4A and Supplementary Figure S3A.

To further optimize screening times, we reduced the hybridization time to 10 min at RT (Figure 4B and Supplementary Figure S3B). The data obtained, as expected, showed a detectable signal in samples that were later identified as being from vaccinated patients or those previously infected by the virus. In contrast, plasma collected prior to any exposure to viral material showed no detectable bands, as shown in Figure 4A,B. The positive outcome observed in the samples hybridized for only 10 min at RT highlights the high potential of the plant-produced antigen as a low-cost and rapid screening tool.

### 2.5. RBDw-Fc Antigen in Plants: A Cheap and Innovative Test

**Figure 5:**
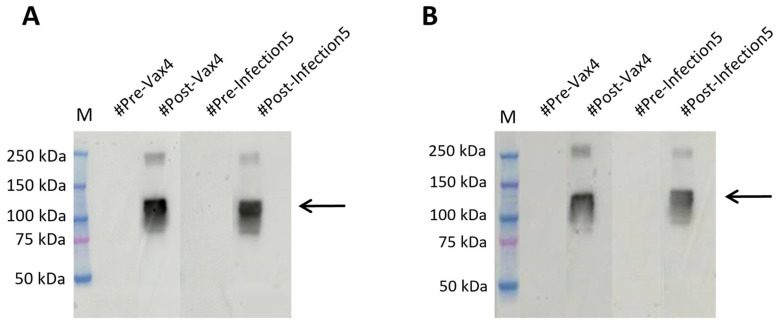
Western blot analysis of SARS-CoV-2 spike protein (RBD), mFc tag recombinant protein (Invitrogen) under non-reducing conditions in plasma EDTA samples. (**A**) The EDTA (#Pre-Vax4 and #Pre-Infection5) showed no signal, and the EDTA (#Post-Vax4 and #Post-Infection5) showed a signal of 110 kDa molecular weight in tests (arrow); overnight (**B**) The EDTA (#Pre-Vax4 and #Pre-Infection5) showed no signal, and the EDTA (#Pre-Vax4 and #Pre-Infection5) showed a signal of 110 kDa molecular weight in rapid test Western blot (arrow).

To corroborate our findings, we compared the results with those obtained by testing the SARS-CoV-2 spike protein (RBD) antigen (aa319-541), mFc tag recombinant protein produced in HEK293 cells (Invitrogen) on the same plasma EDTA samples. Both the overnight hybridization method and the 10 min rapid test were used. We then verified that the antigen recognition was similar to the results previously obtained with our plant-produced antigen (Figure 5 and Supplementary Figure S4).

This result confirms that RBDw-Fc antigen is an innovative, cost-effective, and high-quality test product manufactured from plants.

## 3. Discussion

The recent COVID-19 pandemic, which required large quantities of reagents for high-quality diagnosis delivered quickly and cost-effectively, has drawn the attention of both researchers and pharmaceutical companies to the need for alternative production systems that can meet this growing market demand. In this context, Plant Molecular Farming (PMF) has emerged as a promising strategic candidate.

PMF represents an innovative technological approach that utilizes plants as biofactories to produce bioactive molecules for medical, industrial, and diagnostic applications. As recently reviewed elsewhere [14], compared to traditional systems based on mammalian cell cultures, PMF offers several advantages, including lower production costs, scalability, enhanced and improved biosafety, and a reduced risk of contamination with human or animal pathogens. In particular, transient expression in plants has demonstrated reliability in the generation of vaccine candidates and therapeutic molecules [14]. To date, PMF has proven its efficacy in numerous applications, including the successful production of vaccines against human papillomavirus (HPV), zoonotic viruses, and SARS-CoV-2 during the COVID-19 pandemic [9,15,16,17]. Moreover, plant-derived monoclonal antibodies are increasingly being validated as therapeutic agents [18] and allowing for the low-cost production of industrial enzymes [19]. Finally, plant-produced recombinant antigens are also increasingly prevalent in the development of novel diagnostic tests, as demonstrated in the present study.

Here, we aimed to evaluate a PMF-based strategy for the development and validation of a rapid and low-cost screening method for infectious diseases, using SARS-CoV-2 as our case study. The results obtained not only demonstrate the efficacy of the method developed herein but also its ability to address economic challenges associated with large-scale reagent production.

Most immunological diagnostic tests developed to detect SARS-CoV-2 antibodies rely on the use of the full-length spike glycoprotein, the shorter external S1 segment, the N protein, or the use of the receptor-binding domain (RBD) [20,21]. With regard to the latter, a former study estimated that using *N. benthamiana* plants for the expression of the RBD, between 2 and 4 μg of RBD/g of fresh weight of leaves was obtained [22]. Similar results were achieved by Rattanapisit et al. [23], who produced the RBD domain of SARS-CoV-2 through transient expression in *N. benthamiana*, obtaining 8 μg of this antigen/g of fresh weight of leaves three days after agroinfiltration. In this work, the antigen showed binding specificity for the ACE2 receptor, thus demonstrating its usefulness as a reagent in diagnostics. Further studies have reported even higher yields, of 10 μg/g [20] and 25 μg/g [24], along with the rapid production of different RBD variants of the SARS-CoV-2 spike protein [13,25], demonstrating the suitability of plant-based platforms for serological reagent production in response to future pandemics.

With respect to these previous studies, our work demonstrates significant advancements in antigen yield and purity. Indeed, we have shown the maximum level of accumulation, just two days after agroinfiltration, with a yield of 45.8 ± 15.8 mg per kg of agroinfiltrated leaves. Unfortunately, as previously reported by Fagiani and colleagues [13], the RBDw-Fc recombinant antigen is heavily degraded in plants, and this degradation occurs downstream of the CPPCKC sequence in the hinge region of the mouse Fc as determined by MS analysis. Indeed, this region is highly susceptible to plant proteolytic enzymes and, in particular, to plant cysteine proteases [26,27]. To increase the yield of intact antigen, either the use of the SlCYS8 Cys protease inhibitor [28] during the agroinfiltration process or the specific stabilizing mutagenesis of the hinge region [29] could be used in the future as effective strategies. It must be noted that the yield of purified antigen is a total yield that includes both the intact antigen and the degradation products. Previously reported data [13] quantified the intact RBDw-Fc antigen present in the purified product by ELISA, and the percentage of the intact antigen corresponds to about 11% of the total purified protein. Here, we demonstrated high affinity and binding specificity of our RBDw-Fc antigen versus human IgG. Then, we optimized the amount of antigen requirement to 1 μg and a dilution of 1:2000 for human plasma EDTA, incubated for only 30 min; as a result, we optimized the binding conditions and reduced diagnostic times. Indeed, high antigen concentrations can saturate the membrane and lead to nonspecific binding or a strong background, whereas excessively low concentrations may fail to capture low-abundance antibodies. Therefore, different antigen concentrations were tested to define the minimal amount that still retained robust binding with post-infection or post-vaccination plasma samples. Similarly, plasma dilutions were optimized to balance sensitivity and specificity; more concentrated plasma (e.g., 1:500) could increase the background due to unspecific binding, while higher dilutions (1:2000) provided a cleaner signal without reducing the detection of specific antibodies.

Theoretically, 1 kg of agroinfiltrated leaves would be sufficient to perform about 48,000 tests. A previous study on plant-based biopharmaceutical manufacturing calculated that in about three months, it is possible to obtain sufficient product to enter clinical testing, compared to the six months required when using mammalian cells [30]. Finally, our data show that the RBDw-Fc antigen-based test produced in plants features strong and easy production, high specificity, a short timeframe, and significantly reduced costs, without animal involvement and with greater sustainability. All these features together represent a critical point towards practical applications in clinical and point-of-care settings.

There are, of course, some limitations that must be acknowledged. As mentioned above, the RBDw-Fc antigen degradation during the production process remains challenging. Although we demonstrated high specificity and rapid test performance, the percentage of intact antigen is low, as previously reported [13]. However, promising strategies, such as co-expression with protease inhibitors or hinge region stabilization, may mitigate this issue for future applications. Moreover, the validation of the diagnostic assay was performed on a limited number of biological samples. To mitigate this issue, the validation group included both pre- and post-vaccination or infection plasma in order to consider different conditions in clinical settings. Future studies on enlarged study cohorts are advisable to strengthen the data reported herein. This, in turn, will also allow for the evaluation of batch-to-batch variability and downstream processing optimization for industrial-scale production.

Despite these limitations, our work contributes to the growing body of evidence supporting PMF as an innovative and scalable approach to producing diagnostic kits. Indeed, the flexibility of PMF suggests its potential for rapid adaptation to other infectious diseases and holds promise for addressing current and future global health challenges. Future research could focus on expanding PMF-based diagnostics to include multiplex assays or custom-designed antigens for specific viral variants, further assessing the role of PMF in the field of global diagnostic strategies.

In conclusion, our study highlights that PMF is not only a valid solution for producing antigens for SARS-CoV-2 but also represents an innovative solution that may potentially impact diagnostic reagents production on a global scale.

## 4. Materials and Methods

### 4.1. Plant Production of RBDw-Fc

*N. benthamiana* plants were grown in a hydroponic system on rock wool cubes (Cultilene Grodan, Rijen, The Netherlands) with Hydro Grow type nutrient solution (Growth Technology Ltd., Taunton, UK) at 24 °C under LED lamps (Valoya AP673L, Helsinki, Finland) with light/dark cycles of 16/8 h. Expression was carried out in fucosyl and xylosyltransferase knock-out plants optimized for the protein glycosylation profile by genome editing [31]. Transient expression was performed by vacuum agroinfiltration with a suspension of two different LBA4404 *A. tumefaciens* cultures, respectively containing RBDw-Fc [13] and P19 silencing suppressor constructs [32]. Bacteria were pelleted by centrifugation at 4000× *g*, resuspended in infiltration buffer (10 mM MES, 10 mM MgCl_2_, pH 5.8), and suspensions were mixed (RBDw-Fc and P19 at a 1:1 ratio), reaching a final OD600 of 0.5 for each construct. Plants were infiltrated at the 6–7 leaves stage and grown for another 6 days post-infiltration. For recombinant protein purification, batches of agroinfiltrated leaves (40 g) were collected, frozen in liquid nitrogen, and stored at −80 °C before use.

### 4.2. Analysis of Plant-Produced RBDw-Fc by Western Blotting

Plant tissues were triturated in liquid nitrogen with a mini tissue grinder pestle in an Eppendorf tube by adding an extraction buffer (100 μL/100 mg of tissue) consisting of 1× PBS and a protease inhibitor cocktail (Complete Mini, Roche). Each sample was then centrifuged at 20,000× *g* for 20 min at 5 °C. The supernatant containing total soluble proteins (TSP) was then recovered. The extracts were normalized to each other for the total amount of soluble protein using a Bradford colorimetric assay.

Plant extracts were separated using a 12% T SDS-PAGE acrylamide gel and analyzed by Western blotting. Proteins were electrotransferred onto a PVDF membrane (Trans-Blot Turbo Mini 0.2 µm PVDF Transfer Packs, Bio-Rad, Hercules, CA, USA) using the Trans-Blot^®^ Turbo™ Transfer System (1704150, Bio-Rad, Hercules, CA, USA). Membranes were blocked overnight with PBS containing 4% *v*/*v* milk. For detection of the RBDw-Fc protein, the membrane was incubated with an anti-mouse-Fc HRP KPL (4741802, Milford, MA, USA) conjugated antibody at a dilution of 1:5000 in PBS containing 2% *v*/*v* skimmed milk for 1 h at room temperature (RT). Proteins were detected by enhanced chemiluminescence (ECL™ Prime Western Blotting System, Merck, Darmstadt, Germany) with the Invitrogen iBright CL1500 imaging system (Thermo Fisher Scientific, Waltham, MA, USA).

### 4.3. RBDw-Fc Extraction and Protein-A Affinity Chromatography

Plant tissues frozen in liquid nitrogen were ground into a powder using a pestle and mortar, and 3.5 g of polyvinylpolypyrrolidone (PVPP) was added to 40 g of leaf tissue. An extraction buffer (2 mL/g of leaves) consisting of PBS containing a protease inhibitor cocktail (Complete TM Roche, Basel, Switzerland) and ascorbic acid (0.25 g in 80 mL) was added; the resulting mixtures were homogenized with an Ultra-Turrax homogenizer T25 (IKA, Staufen, Germany). The slurry was filtered through Miracloth with a pore size of 22–25 µm (Sigma-Aldrich, St. Louis, MO, USA) and clarified by double centrifugation at 8000× *g* for 20 min at 4 °C. The supernatant was loaded onto a protein-A affinity column (1 mL HiTrap™ Protein-A FF, GE Healthcare, Chicago, IL, USA) at a flow rate of 1 mL/min. The column was washed with 10 vol of PBS, and each antibody was eluted with 200 mM Tris-HCl, 100 mM glycine, pH 3.0. Eluted fractions (0.5 mL each) were neutralized to about pH 7.0 with 100 mL of 1 M Tris-HCl pH 9.5. The eluted proteins were dialyzed/concentrated in PBS by ultrafiltration with a Vivaspin^®^ 5000 MWCO HY concentrator (Sigma-Aldrich, Burlington, MA, USA). Total purified antigen concentration was determined by measuring the absorbance at 280 nm on a spectrophotometer, and the corresponding purity was evaluated by SDS-PAGE followed by Coomassie blue staining.

### 4.4. Biological Samples Analyzed

For the analysis of the spike SARS-CoV-2 protein, a total of 110 biological samples from 95 subjects were used. These included 3 plasma EDTA, 3 sera, 3 plasma lithium heparin, and 3 plasma citrate positive samples obtained from 3 patients with a confirmed diagnosis of COVID-19 used as controls, as well as 98 plasma EDTA blind samples, all collected from the Interinstitutional Multidisciplinary BioBank (BioBIM) of the IRCCS San Raffaele in Rome, Italy, as described in Supplementary Table S1.

Anonymous samples were provided from patients recruited over the years. In particular, the screened samples included both samples taken prior to the COVID-19 pandemic, from January 2016 to December 2018, and follow-up samples taken after vaccine inoculation or the viral infection, from January 2021 to December 2022, thus allowing for results comparison between the baseline and post-vaccine or post-viral infection samples. All participants were recruited and followed up under appropriate institutional ethics approval and in accordance with the principles of the World Medical Association Declaration of Helsinki. All activities related to this project were performed in accordance with the national regulations of the EU General Data Protection Regulation (GDPR) (Reg. EU 2016/679) and the EU Charter of Fundamental Rights, both with respect to the security and protection of personal data and the technical requirements for storing these data.

Each of the patients previously signed an informed consent for data processing and privacy protection according to GDPR and current Italian (Art. 13 of Legislative Decree no. 196/2003) regulations.

### 4.5. Western Blot Analysis of Blood Samples

Three different batches of the plant extracts RBDw-Fc produced in *Nicotiana benthamiana* (1.0 µg/µL, 1.4 µg/µL, and 1.7 µg/µL) [13] were prepared with a 4× Laemmli sample buffer (Bio-Rad, Hercules, CA, USA), boiled at 95 °C for 5 min, and run on a non-reducing 4–20% gradient SDS-PAGE (Mini-Protean TGX Stain-Free Precast Gels, Bio-Rad, Hercules, CA, USA). For Western blot analysis, proteins were transferred to a nitrocellulose membrane (Trans-Blot Turbo Mini 0.2 µm Nitrocellulose Transfer Pack, Bio-Rad, Hercules, CA, USA) with a Transfer unit (Trans-Blot^®^ Turbo™ Transfer System, Bio-Rad, Hercules, CA, USA) for 7 min. Membranes were blocked in 10 mL of EveryBlot Blocking Buffer (Bio-Rad, Hercules, CA, USA) for 5 min, with agitation at RT, and washed three times with TBS-T (final concentration TBS 1×, cat#1706435; 0.01% Tween-20, cat#1610781, Bio-Rad, Hercules, CA, USA). After this, for the rapid tests, the membranes were incubated with primary antibodies (plasma EDTA 1:500, 1:1000, and 1:2000 in TBS-T containing 5% milk) for 10 min at RT with agitation. The same samples and concentrations of plasma EDTA were incubated overnight at 4 °C, as well as the samples of plasma citrate, plasma lithium heparin, and sera concentrated to 1:500 in TBS-T containing 5% milk. The membranes were washed three times with TBS-T, followed by incubation with horseradish peroxidase-conjugated goat anti-mouse IgG secondary antibody in a dilution of 1:10,000 in TBS-T (Invitrogen cat#31482, Waltham, MA, USA) for 10 min with agitation in a rapid test and 1 h for normal analysis. After washing, the signal was detected using LiteAblot ^®^PLUS Enhanced Chemiluminescent Substrate for Western Blotting (Euroclone S.p.A., Milan, Italy) and imaged using Amersham ImageQuant™ 800 (Amersham, UK). The protein molecular weight markers were BenchMark™ Pre-stained Protein Ladder (Invitrogen, Waltham, MA, USA) and Precision Plus Protein Kaleidoscope (Bio-Rad, Hercules, CA, USA).

## Data Availability

The data used to support the findings of this study are provided within this article. Further information can be provided by the corresponding author upon request.

## Supplementary Materials

The following supporting information can be downloaded at https://www.mdpi.com/article/10.3390/ijms26104500/s1.
