# Supplementary material for: Rapid and Cost-Effective Diagnostic Blot Assays Based on the Use of Plant-Produced Recombinant Antigens: Lessons Learned from the SARS-CoV-2 RBD Antigen
Source: Int J Mol Sci. 2025 May 8;26(10):4500. doi: 10.3390/ijms26104500 (PMC12111317; doi:10.3390/ijms26104500)
Supplement: Supplementary file 1 [file ijms-26-04500-s001.zip › ijms-3615685-supplementary.pdf]

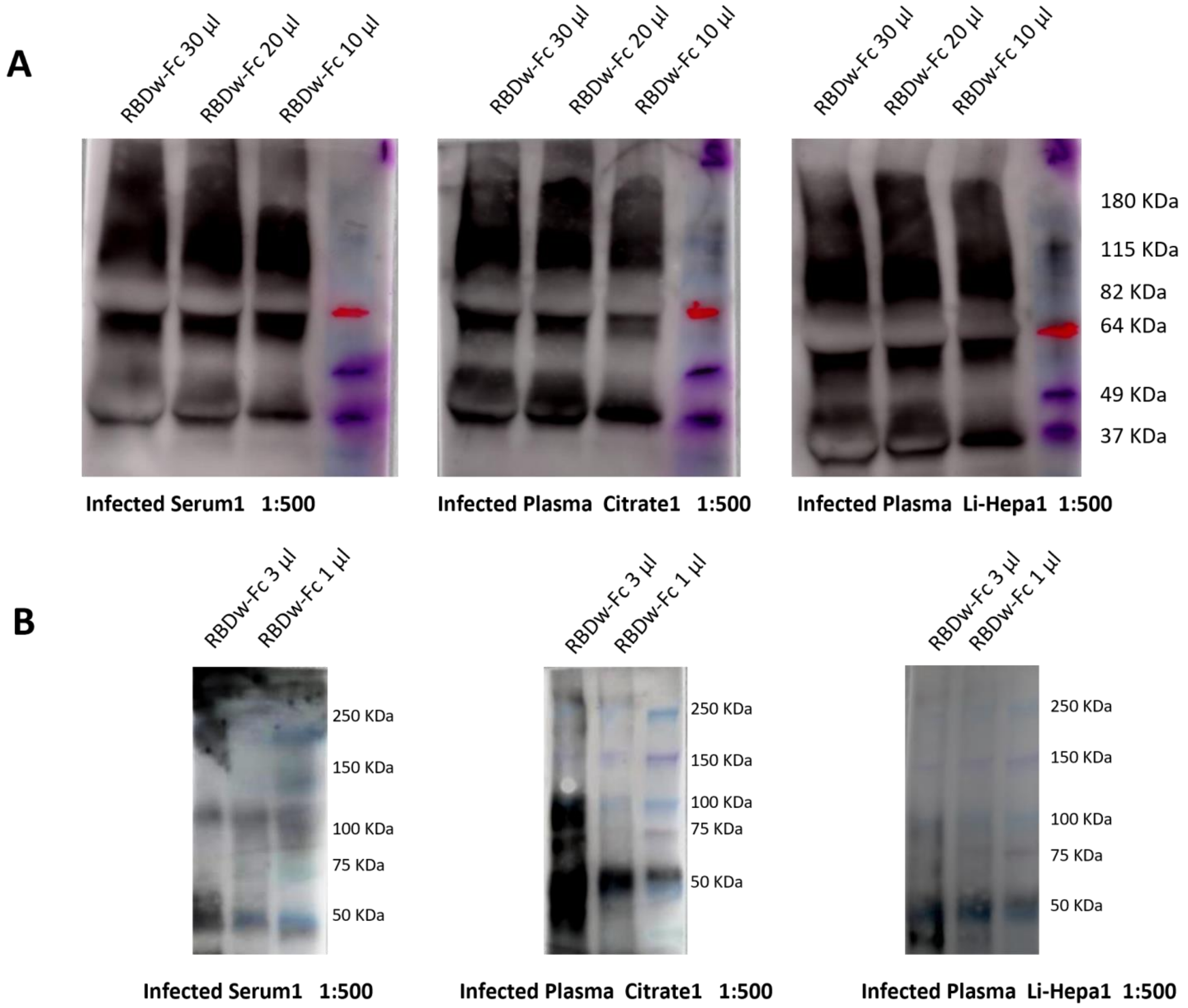

Supplementary Figure S1: (A) 30 µg/µl, 20 µg/µl, 10 µg/µl of RBDw-Fc antigen were hybridized with paired post infection known serum (left panel A), plasma citrate (central panel A) and plasma Lithium Heparin (right Panel A) samples 1:500 in milk 5%. Loading Marker (M) BenchMark™ Pre-stained Protein Ladder. (B) 3µg/µl and 1µg/µl of RBDw-Fc antigen were hybridized with paired post infection known serum (left panel B), plasma citrate (central panel B) and plasma Lithium Heparin (right panel B) 1:500 in milk 5%. Loading Marker (M) Kaleidoscope Bio-Rad.

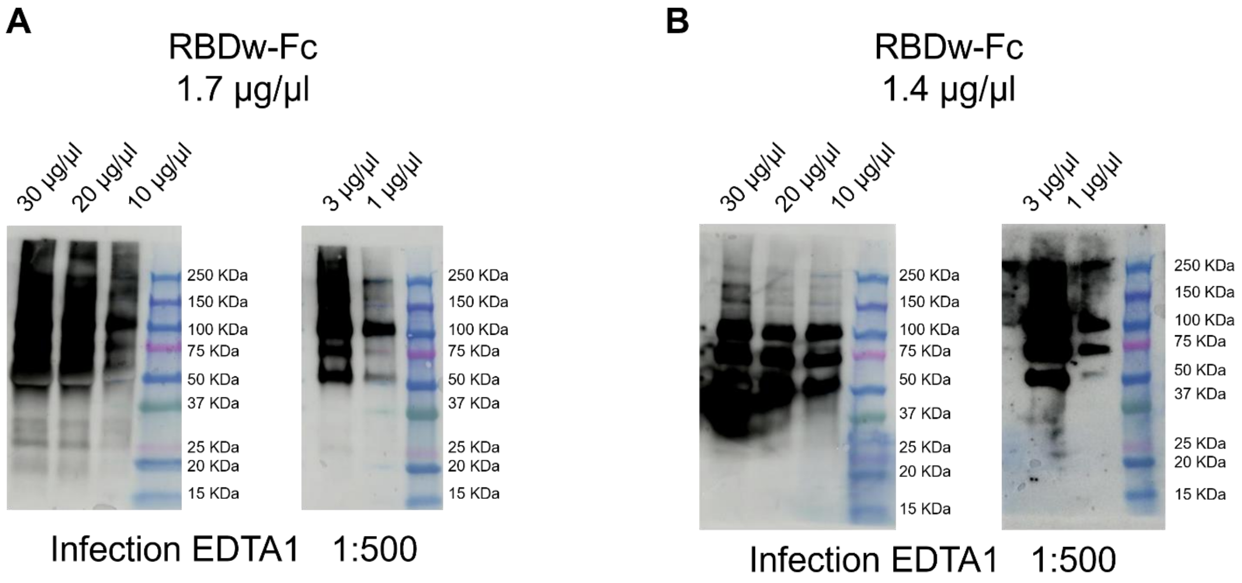

Supplementary Figure S2: (A) 30 µg/µl, 20 µg/µl, 10 µg/µl (left panel A), 3µg/µl and 1µg/µl (right panel A) of RBDw-Fc 1.7 µg/µl antigen were hybridized with a post infection known EDTA plasma sample 1:500 in milk 5%. Loading Marker (M) Kaleidoscope Bio-Rad. (B) 30 µg/µl, 20 µg/µl, 10 µg/µl (left panel B), 3µg/µl and 1µg/µl (right panel B) of RBDw-Fc 1.4 µg/µl antigen were hybridized with a post infection known EDTA plasma sample 1:500 in milk 5%. Loading Marker (M) Kaleidoscope Bio-Rad.

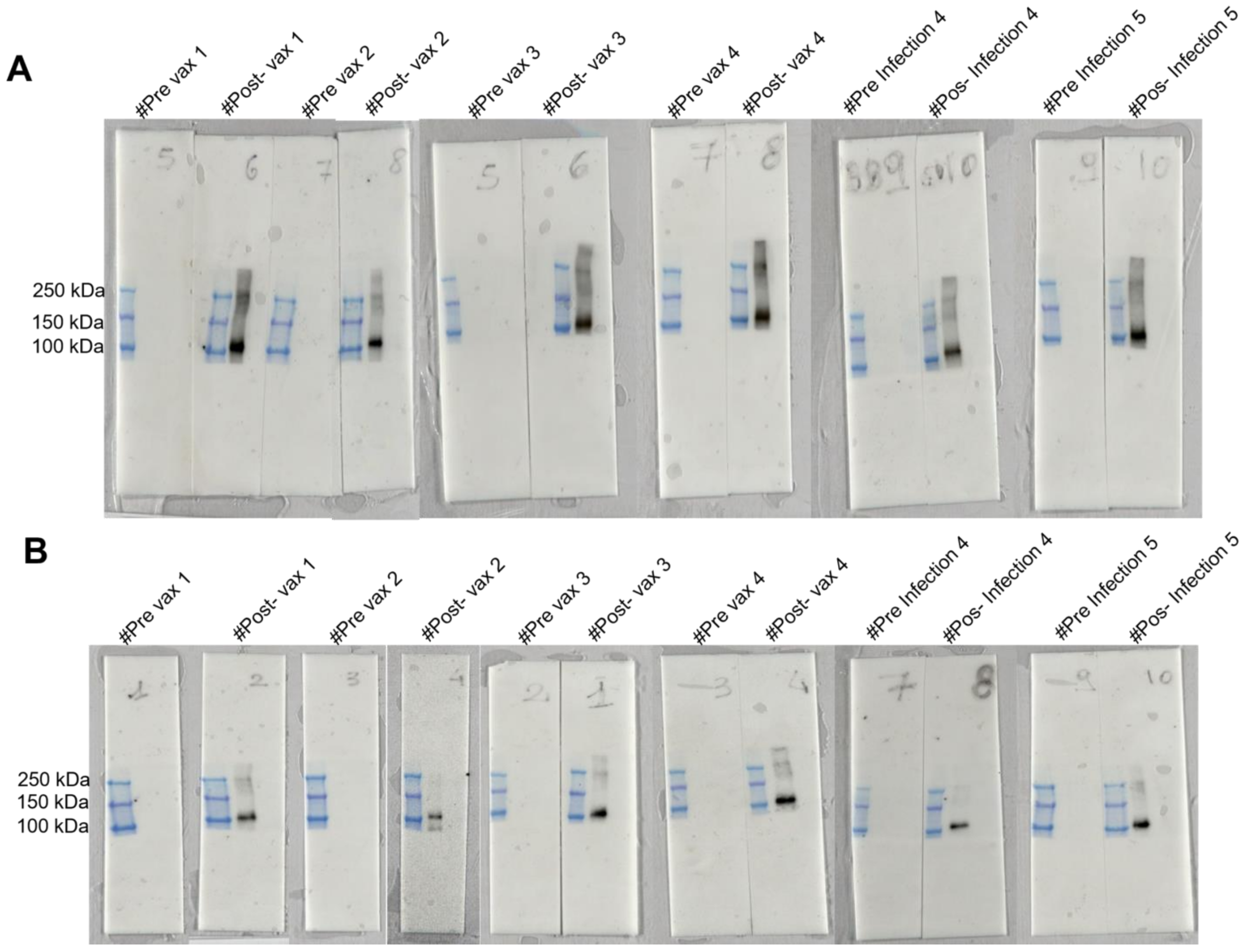

Supplementary Figure S3: representative uncropped blots from negative (pre) and positive (post) EDTA samples shown in Figure 4. Loading Marker Kaleidoscope Bio-Rad. Panel A: overnight incubation; Panel B: rapid test.

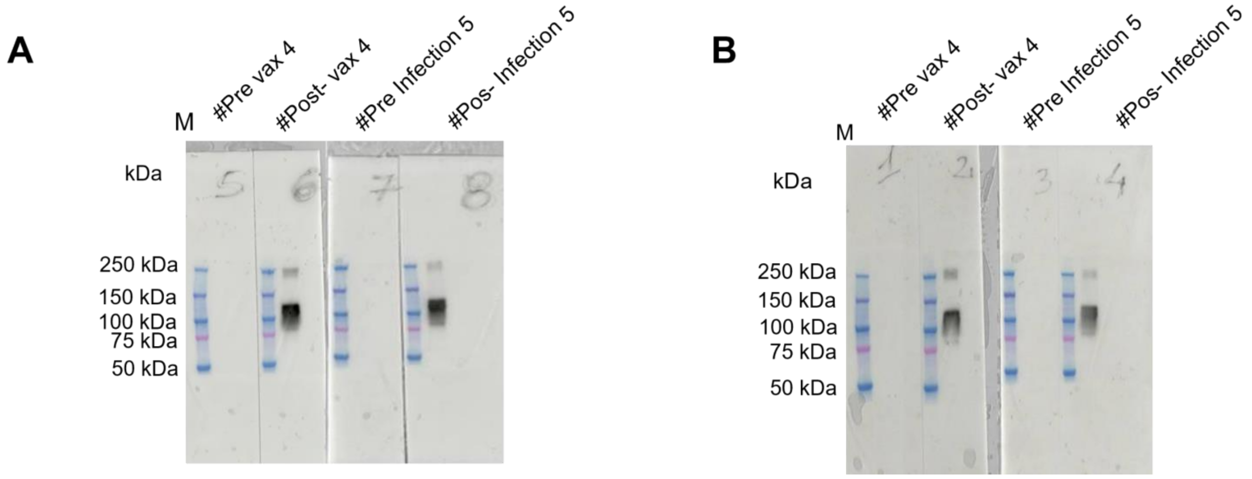

Supplementary Figure S4: representative uncropped blots from negative (pre) and positive (post) EDTA samples shown in Figure 5. Western blot analysis of SARS-CoV-2 Spike Protein (RBD), mFc Tag Recombinant Protein (Invitrogen) under non-reducing condition. Loading Marker Kaleidoscope Bio-Rad. Panel A: overnight incubation; Panel B: rapid test.

Supplementary Table S1: Summary of subjects and samples used for the affinity binding study of SarsCoV2 RBDw-Fc antigen.

110 Samples	95 Subjects	Sampling Period	Know	Blind	RDBw-Fc Test
12 Samples<br>➢ 3 Sera<br>➢ 3 Plasma Citrate<br>➢ 3 Plasma Lithium Heparin<br>➢ 3 Plasma EDTA	3 Patients	From January 2021 to December 2022	Yes	No	Positive
86 Plasma EDTA	38 Subjects	From January 2016 to December 2018	No	Yes	Negative
	48 Subjects [1]	From January 2021 to December 2022	No	Yes	Positive
12 Plasma EDTA	6 Matched Subjects	From January 2016 to December 2018	No	Yes	Negative
		From January 2021 to December 2022	No	Yes	Positive

[1] These samples were obtained from subjects either post-vaccination or post-infection.
